# IMPACT OF CALORIE RESTRICTION ON PLASMA ALZHEIMER’S DISEASE BIOMARKERS IN HEALTHY YOUNG AND MIDDLE-AGED ADULTS

**DOI:** 10.1093/geroni/igac059.388

**Published:** 2022-12-20

**Authors:** Daniel Parker, P Murali Doraiswamy, William Kraus, Kim Huffman

**Affiliations:** Duke University School of Medicine, Durham, North Carolina, United States; Duke University School of Medicine, Durham, North Carolina, United States; Duke University School of Medicine, Durham, North Carolina, United States; Duke University School of Medicine, Durham, North Carolina, United States

## Abstract

Midlife cardiometabolic risk factors are associated with an increased risk of Alzheimer’s dementia (AD). Moderate calorie restriction (CR) in healthy, non-obese young and middle-aged adults improves cardiometabolic risk factors. Plasma concentrations of amyloid β oligomers (Aβ-42 and Aβ-40) and total tau are emerging biomarkers of AD pathology. Our objective was to determine the impact of two years of CR in healthy young and middle-aged adults on Aβ-42, Aβ-40, and total tau in the Comprehensive Assessment of Long term Effects of Reducing Intake of Energy (CALERIE) Study. Participants were randomized 2:1 to 24 months of CR (prescribed as 25% reduction in baseline calorie requirements) versus an ad libitum (AL) diet. We quantified plasma Aβ-42, Aβ-40, and total tau using the ultrasensitive single molecule array (SIMOA) technology at baseline and two years in a subset of CALERIE (N=133). We used linear regression to evaluate the impact of CR, including age, sex, and presence/absence of the APOE-ε4 risk allele as covariates. We hypothesized that there would be differential CR effects based on APOE-ε4 carrier status; to test this, we included an interaction term. As compared to AL, there was a trend towards decreased Aβ-40, controlling for age, baseline Aβ-40 concentrations, and APOE-ε4 carrier status (β=-12.59, 95% CI[-27.14, 1.96], p=0.093) with 12% (average achieved) CR. The CR*APOE-ε4 carrier status interaction term was significant at a pre-defined threshold of p<0.10 (p=0.062). Stratified by APOE-ε4 carrier status, CR was associated with a decrease in plasma Aβ-40 (β=-33.72, 95% CI[-65.16,-2.09], p=0.041). In conclusion, moderate CR in healthy, non-obese young and middle-aged adults impacts plasma biomarkers of AD risk, primarily in APOE-ε4 carriers.

